# QTL mapping for micronutrients concentration and yield component traits in a hexaploid wheat mapping population

**DOI:** 10.1016/j.jcs.2019.05.008

**Published:** 2019-07

**Authors:** Jia Liu, Bihua Wu, Ravi P. Singh, Govindan Velu

**Affiliations:** a Triticeae Research Institute, Sichuan Agricultural University, Wenjiang, 611130, China; b International Maize and Wheat Improvement Center (CIMMYT), Apdo Postal 6-641, Mexico DF, 06600, Mexico

**Keywords:** Wheat biofortification, QTL mapping, Grain mineral nutrient concentration, Agronomic-related traits, GZnC, Grain zinc concentration, GFeC, Grain iron concentration, GPC, Grain protein concentration, TKW, Thousand kernel weight, PH, Plant height, HD, Heading date, MD, Maturity date, QTL, Quantitative trait loci, PVE, Phenotypic variation explained

## Abstract

Bread wheat is a major staple cereal provides more than 20% of dietary energy and protein supply to global population. However, with increasing population growth, the problem of nutritional deficiencies is increasingly affecting the health of resource people with predominantly cereal-based diet. Therefore, the development of wheat genotypes with micronutrient-dense grains along with high-yield potential is one of the major priorities of wheat biofortification program at CIMMYT. We conducted a QTL mapping study using a recombinant inbred line (RIL) population derived from a cross between a Chinese parental line with highGZnC and a Mexican commercial bread wheat cultivar Roelfs F2007 to identify QTLs that could potentially be integrated in mineral nutrient concentrations and agronomic-related traits breeding. We evaluated 200 RIL lines for mineral nutrient concentrations and agronomic-related traits over two years. A total of 60 QTLs were detected, of which 10 QTLs for GZnC, 9 for GFeC, 5 for GPC and 36 for agronomic-related traits. Moreover, a total of 55 promising candidate genes were identified from the list of associated markers for GFeC and GZnC using the recently annotated wheat genome sequence. We identified the promising genomic regions with high mineral nutrient concentrations and acceptable yield potential, which are good resource for further use in wheat biofortification breeding programs.

## Highlights

• Rich genetic diversity for micronutrients in wheat landraces provides novel alleles.
• QTLs identified for multiple traits offers breeding competitive biofortified wheat.
• Genomic regions and the candidate genes identified enables marker assisted breeding.

## 1 Introduction

Poor quality diets dominated by food staples, are often deficient in minerals and vitamins, causing mineral malnutrition problem or ‘hidden hunger’, which affects more than 2 billion people globally. Especially iron (Fe) and zinc (Zn) deficiencies cauing serious health problems in pregnant women and childrens below 5 years of an developing regions. Anemia, whose largest cause is Fe deficiency, afflicts 24.8% of the global population and more than 65% of the preschool-age children in Africa and South-east Asia (HO, 2008). Besides, Fe deficiency is the 13th most significant global health risk factor in 2010, accounting for 48,225,000 disability-adjusted life years (Murray and Lopez, 2013). Meanwhile, inadequate Zn intake threatens estimated 17.3% of the global population, which mostly distributes in Asian and African developing countries (Wessells and Brown, 2012). In addition, Zn deficiency is responsible for the annual mortality of 433,000 children under the age of five (WHO, 2009).

One of the measures to alleviate Fe and Zn deficiencies is to increase the GFeC and/or GZnC within edible parts of agricultural crops, which is named asbiofortification. Among various means to tackle Fe/Zn deficiencies (Stein, 2010), breeding high-GFeC/GZnC crops through genetic biofortification has emerged as a most cost-effective and sustainable approach. Although being a poor source of micronutrients such as Fe and Zn, cereals are dominant in the diet of most resource-scarce population in developing regions (Graham et al., 2001). Wheat (*Triticum aestivum*) is the cereal with the third largest global production after maize and rice (FAO, 2014), contributing to 17% of the calories in the human diet in the developing world (Braun et al., 2010). Hence, biofortification of wheat by means of breeding is a promising strategy to ameliorate Fe and Zn deficiencies in the developing countries, and therefore increasing the GFeC/GZnC has been an important breeding target in the recent wheat breeding programs.

Modern wheat varieties have limited variation for grain Zn and Fe, hence a large scale screening of wheat genetic resources at the germplasm bank of the International Maize and Wheat Improvement Center (CIMMYT) showed large variation for Zn and Fe amongst the wild relatives and landraces (Ortiz-Monasterio et al., 2007; Velu et al. 2011, 2019). The available genetic variation from different species and landraces were utilized in the biofortification breeding program to develop nutrient-enriched wheat germplasm with competitive yield potential and stress tolerance (Velu et al., 2014).

While GFeC and GZnC biofortification is an important objective in wheat breeding programs, conventional traits such as grain yield and GPC should not be compromised in exchange of mineral concentrations as the farmers income primarily depends on the grain yield. In addition, wheat is a major source of protein accounting for 20% of human protein intake in the developing regions (Braun et al., 2010). As been reviewed by Cakmak et al. (2000), there is generally no or only a slight negative correlation between the yield and GFeC/GZnC. However, recent research by Velu et al. (2016) involving advanced lines under heat and drought stresses showed a significant negative correlation between the GZnC and grain yield per unit area, indicating that breeding for high GZnC would not optimize the yield under such stress conditions. Nonetheless, it has been reported that protein concentration of the wheat grain decreases under elevated air CO_2_ concentration of 550 μmol/mol (Fernando et al., 2012). By the end of the 21^st^ century, it is predicted that the global temperature will rise by 1.1–3.1 °C and atmospheric CO_2_ concentration will reach above 550 ppm under intermediate scenarios (IPCC, 2014). Moreover, it is expected that heat waves will occur with a higher frequency and longer duration (IPCC, 2014). Given these climatic variations predicted in the coming decades and how wheat grain yield and GPC would be affected by such changes, it is paramount to improve grain yield potential and stress tolerance with additional levels of GFeC and GZnC in the biofortification breeding. Therefore, identifying the quantitative trait loci (QTL) that regulate the accumulation of high mineral nutrient concentration in the wheat grain and yield related agronomic traits would allow breeders to develop biofortified cultivars more efficiently by using closely linked molecular markers to screen and select the most favourable genotypes.

In this study, a recombinant inbred line (RIL) population derived from a cross between a Mexican commercial bread wheat cultivar (Roelfs F2007) and a highGZnC cultivar with Chinese origin was developed. The main objective of the study was to: (a) explore the genotypic potential of a biofortified wheat cultivar with Chinese progenitor in its background for GFeC, GZnC and GPC and grain yield; (b) identify genetic loci associated with high GFeC, GZnC, GPC and yield component traits to provide evidence for breeding biofortified wheat varieties with high mineral nutrient concentration and TKW; and (c) determine the relationship between mineral nutrients concentration and other associated agronomic traits.

## 2 Materials and methods

### 2.1 Plant materials

An F6 recombinant inbred lines (RIL) population between a Mexican commercial bread wheat cultivar Roelfs F2007 as female and a Chinese parental line whose lineage is Hong Hua Mai/. ../Blouk #1 consisting of 200 recombinant lines were grown at Norman E. Borlaug Research Station, Ciudad Obregon, Sonora, Mexico along with their parental lines. Hong Hua Mai, which is one of the Chinese landrace previously reported to have high GFeC/GZnC.

### 2.2 Phenotyping and analysis of phenotypic data

The RIL population was evaluated forGZnC, GFeC, GPC concentrations and other agronomic traits in Ciudad Obregon, Mexico (Norman E. Borlaug Experiment Station) for two successive crop seasons during the 2016–2017 (Y16-17) and 2017–2018 (Y17-18). Each entry was grown on a double-row plot of 1 m long and 0.8 m wide in a bed-planting system; a randomized complete block design with two replicates was utilized to conduct the evaluations. Diseases and pests were controlled chemically, whereas weeds were controlled manually and chemically. Plant materials were harvested after physiological maturity when grains were totally dry in the field. Grain samples of about 20 g for each entry were carefully cleaned to discard broken grains and foreign material, and used for micronutrient and protein analysis. Mineral concentrations in the grains (GZnC and GFeC) were measured with a ‘bench-top’, non-destructive, energy-dispersive X-ray fluorescence spectrometry (EDXRF) instrument (model X-Supreme 8000, Oxford Instruments plc, Abingdon, UK) that was standardized for high-throughput screening of GZnC and GFeC (unit: mg/kg) content in whole grain wheat (Paltridge et al., 2012). About three laboratory commercial checks (PBW343, Borlaug100 F2014 and Kachu #1) were used as in-house quality control checks. These checks were chosen to ensure wide range of GZnC and GFeC in wheat samples. The XRF machine at CIMMYT, Ciudad Obregon, Mexico showed within the acceptable levels of accuracy in a proficiency study conducted by Flinders University, Adelaide by comparing XRF data with the Inductively Coupled Plasma (ICP) results and confirmed that XRF data is reproducible and as accurate as ICP results. GPC was assessed using near-infrared reflectance (NIR Systems 6500, Foss Denmark) according to the official method AACC 39–70A.

Thousand-kernel weight (TKW) were measured with SeedCount digital imaging system (model SC5000, Next Instruments Pty Ltd, New South Wales, Australia) that was standardized to measure TKW (g 10^−3^ kernels). The Seed-Count system can rapidly and accurately analyze wheat grain samples, determining the grain number and grain physical characteristics based on software and flatbed scanner technology. The data on days to maturity (MD) were measured in Y16-17 (E1) and Y17-18 (E2) environments by counting the number of days from germination to physiological maturity when more than 50% of spikes were ripe and had turned yellow. The data on days to heading (HD) were measured in Y16-17 (E1) and Y17-18 (E2) environments by counting the number of days from germination to physiological heading. Plant height (PH) was measured from the ground to the tip of the spike excluding awns at the late grain-filling stage.

Analyses of variance (ANOVA) and pearson correlation coefficients for traits in each year were performed using the SPSS version 22.0 for Windows (SPSS Inc., Chicago, IL, USA).

### 2.3 Genotyping, linkage mapping and QTL analysis

DNA was extracted according to standard procedures (Dreisigacker et al., 2013) and genotyping was done with DArT sequence at CIMMYT, SAGA platform. The genotypic information consisted of an initial number of 45021 markers codified as 0 and 1 for homozygote and 2 for heterozygote. Parental non-polymorphic markers were discarded. Thus, a total of 3556 DArT markers were used for the QTL analyses.

The linkage map was assembled from the genotypic data using QTL IciMapping v3.2 software (http://www.isbreeding.net), applying a LOD threshold of 3.0 between adjacent markers (Li et al., 2007). QTLs were identified with the inclusive composite interval mapping (ICIM) algorithm for additive gene effects implemented in QTL IciMapping v.3.2 software. The QTL expressed in each environment were defined, as well as set of QTL which were stable across all the environments. For both procedures, the walking step was set to 1 cM and a relaxed LOD threshold of 2.5 was applied to call significance. QTL nomenclature was standard (http://wheat.pw.usda.gov/ggpages/wgc/98/Intro.htm).

## 3 Results

### 3.1 Phenotypic evaluations

Mean values of traits for the parents ‘Roelfs F2007’ and Chinese parental line, and for the recombinant inbred lines (RIL) over environments are shown in Table 1. Large differences between the parents were observed for GZnC and plant height traits, whereas small differences between the two parents were found for GFeC, GPC, TKW, HD and MD. Mineral nutrient concentrations and agronomic traits in the RIL population showed wide range of variation. These results indicated that RIL population showed transgressive segregation, suggesting polygenic inheritance of the traits. In all environments, the mean value of GZnC and GFeC was higher than that of both parents and three commercial checks (Table 1). Among them, the highest mean GZnC (89.10 mg/kg) was recorded in E2 environment, whereas the lowest mean for GZnC (39.60 mg/kg) was recorded in E1 environment and the highest GFeC (63.10 mg/kg) was also recorded in E1. In addition, the mean value of GPC was higher than that of both parents and PBW343, whereas the mean value of plant height was lower than that of both parents. For the other agronomic trait, there was no significant difference between both parents and RILs mean value in all environments (Table 1).

**Table 1:** Phenotypic values of each traits of RILs, parents and three commercial checks evaluated during 2016 and 2018 at Ciudad Obregon, Mexico.

Environment	Trait	Roelfs F2007	Chinese parental line	RIL			PBW343	BORLAUG100 F2014	KACHU #1
		Mean ± SD	Mean ± SD	Range	Mean ± SD	CV (%)	Mean ± SD	Mean ± SD	Mean ± SD
Ciudad Obregon Y16-17 (E1)	GFeC (mg/kg)	37.00 ± 0.14	38.50 ± 0.28	32.00–63.10	41.76 ± 4.64	11.11	34.20 ± 2.12	40.60 ± 1.13	38.20 ± 1.84
	GZnC (mg/kg)	37.30 ± 0.71	45.90 ± 0.14	39.60–66.90	52.64 ± 4.58	8.70	47.05 ± 0.07	47.40 ± 0.28	43.80 ± 0.00
	GPC (%)	13.30 ± 0.00	12.85 ± 0.35	11.80–16.90	13.79 ± 0.69	5.00	13.25 ± 0.07	14.35 ± 0.35	14.30 ± 0.00
	TKW (g)	44.80 ± 0.00	45.30 ± 0.42	36.90–56.40	45.59 ± 3.57	7.83	47.05 ± 0.21	47.65 ± 0.07	42.25 ± 1.91
	PH (cm)	116.00 ± 0.00	102.00 ± 7.07	86.00–115.00	100.88 ± 4.64	4.60	91.50 ± 4.95	96.00 ± 1.41	94.50 ± 3.54
	HD (day)	82.00 ± 0.00	78.00 ± 0.00	66.00–98.00	82.84 ± 5.61	6.77	85.00 ± 0.00	78.00 ± 0.00	79.00 ± 0.00
	MD (day)	121.00 ± 0.00	121.00 ± 1.41	108.00–132.00	123.32 ± 3.22	2.61	123.50 ± 0.71	121.00 ± 1.41	122.00 ± 0.00
Ciudad Obregon Y17-18 (E2)	GFeC (mg/kg)	42.40 ± 0.57	40.60 ± 1.56	36.10–58.30	45.62 ± 3.61	7.91	44.65 ± 5.16	43.35 ± 3.89	41.90 ± 3.54
	GZnC (mg/kg)	56.10 ± 1.84	65.55 ± 8.41	50.40–89.10	66.88 ± 6.59	9.85	63.65 ± 8.56	60.80 ± 8.63	55.85 ± 4.74
	GPC (%)	12.50 ± 0.00	12.25 ± 0.07	11.40–16.50	13.36 ± 0.72	7.49	12.95 ± 0.07	14.05 ± 0.21	14.00 ± 0.14
	TKW (g)	52.55 ± 0.35	50.45 ± 1.63	38.90–62.90	49.25 ± 4.19	8.51	49.05 ± 1.06	52.10 ± 0.28	47.85 ± 0.49
	PH (cm)	99.50 ± 0.71	96.50 ± 2.12	80.00–113.00	95.29 ± 5.34	5.60	87.50 ± 0.71	88.50 ± 0.71	88.00 ± 0.00
	HD (day)	80.00 ± 0.00	81.00 ± 0.00	73.00–98.00	83.02 ± 3.48	4.19	87.00 ± 0.00	80.00 ± 0.00	79.00 ± 0.00
	MD (day)	129.00 ± 2.83	126.00 ± 0.00	116.00–140.00	127.72 ± 2.75	2.15	128.50 ± 2.12	125.50 ± 0.71	126.50 ± 0.71
Pooled ((mean of two environments (E3))	GFeC (mg/kg)	39.70 ± 3.82	39.55 ± 1.48	37.60–52.13	43.69 ± 3.64	8.33	39.43 ± 7.39	41.98 ± 1.94	40.05 ± 2.62
	GZnC (mg/kg)	46.70 ± 13.29	55.75 ± 13.93	50.10–69.28	59.78 ± 8.37	14.00	55.38 ± 11.77	54.10 ± 9.48	49.85 ± 8.56
	GPC (%)	12.90 ± 0.57	12.55 ± 0.42	11.80–16.20	13.58 ± 0.67	4.93	13.10 ± 0.21	14.20 ± 0.21	14.15 ± 0.21
	TKW (g)	48.70 ± 5.52	47.90 ± 3.68	38.60–58.13	47.43 ± 4.25	8.96	48.08 ± 1.45	49.88 ± 3.15	45.08 ± 4.00
	PH (cm)	107.75 ± 11.67	99.15 ± 4.03	87.75–107.63	97.98 ± 5.44	5.55	89.40 ± 2.97	92.15 ± 5.44	91.25 ± 4.60
	HD (day)	81.00 ± 1.41	79.50 ± 2.12	69.50–98.00	82.93 ± 4.66	5.62	86.00 ± 1.41	79.00 ± 1.41	79.00 ± 0.00
	MD (day)	125.00 ± 5.66	123.50 ± 3.54	112.50–134.25	125.52 ± 3.55	2.83	126.00 ± 3.54	123.25 ± 3.18	124.25 ± 3.18

**Fig. 1:**
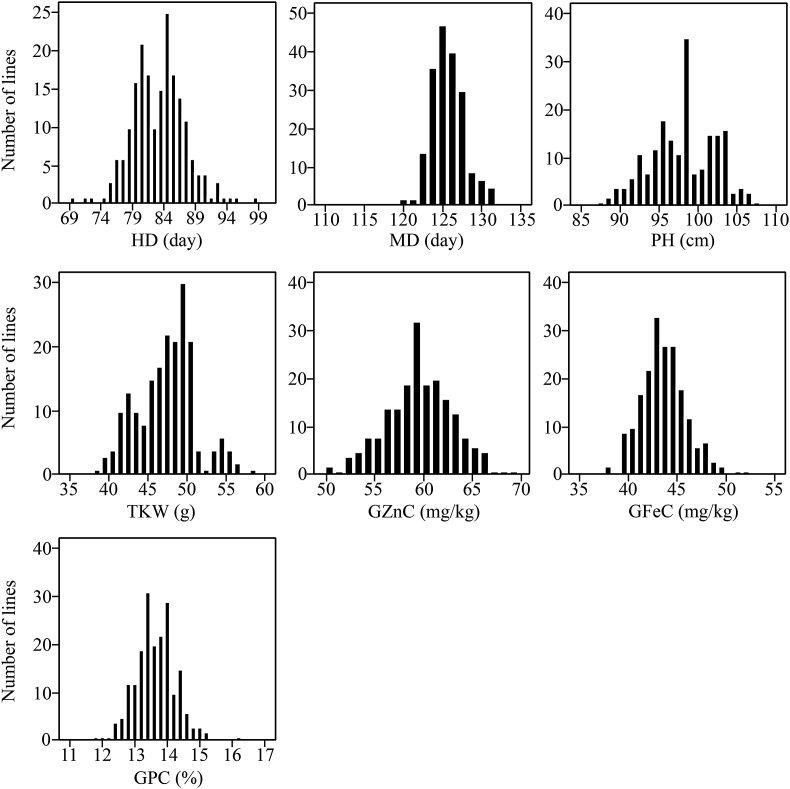
Frequency distribution of seven traits: mean performance over two environments of the 200 RILs.

The correlation coefficients between two mineral nutrient traits, GPC and four agronomic traits were calculated (Table 2). For the mineral nutrient traits, the strongest positive correlation (r = 0.517; P < 0.01) was observed between GZnC and GFeC. Moreover, slight negative correlations were found between PH and TKW (r = –0.032), and between PH and GZnC (r = –0.009), but were not significant. HD showed a significant positive correlation with MD and PH, but a negative correlation with TKW. There was a significant positive correlation between MD and PH. Significant negative correlation were also found between MD and TKW. Both GZnC and TKW showed a significant positive correlation with GPC. However, both GPC and TKW showed no significant correlation with GFeC. (Table 2). The distribution of traits across two years was continuous (Fig. 1). The highest frequency distribution region of each trait was different, such as the GZnC was about 60 mg/kg, GFeC was about 43 mg/kg, GPC was around 13–14%, HD was around 84 days, MD was 125 day, PH was 99 cm and TKW was 50 g.

**Table 2:** Pairwise correlation coefficients among traits from RIL lines across two years.

Correlations	HD	MD	PH	TKW	GZnC	GFeC
MD	0.831**					
PH	0.464**	0.334**				
TKW	−0.463**	−0.528**	−0.032			
GZnC	0.088	0.014	−0.009	0.115		
GFeC	0.170*	0.116	0.159*	−0.133	0.517**	
GPC	−0.115	−0.118	−0.106	0.190**	0.348**	0.076

*significant r-values p < 0.05, ** significant r-values p < 0.01.

### 3.2 Construction of genetic map

The linkage map was constructed using 3556 informative markers. A total of 1198 marker mapped to A genome chromosomes, 1955 to B genome chromosomes and 403 to D genome chromosomes. Among them, the 1B chromosome has the most markers (520), and the 3D chromosome has the least markers (41) (Table S1). The full map covered a genetic length of 5747 cM with a mean inter-marker distance of 1.6 cM.

### 3.3 QTL analysis

**Fig. 2:**
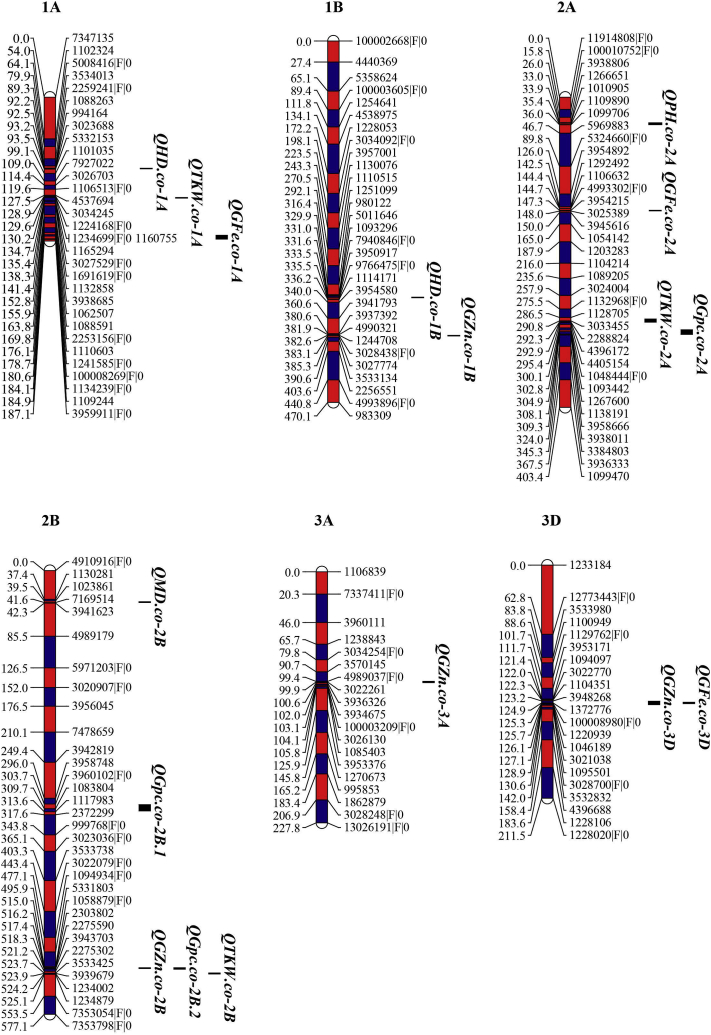
Sixty QTLs for mineral nutrient concentrations and agronomic-related traits on the genetic map of the ROELFS F2007/Chinese parental line derived RIL population. Mapped markers are indicated on the right and their corresponding genetic distances (cM) are indicated on the left.

QTLs detected by ICIM were shown in Table 3, and their map positions provided in Fig. 2. A total of 60 QTLs were detected in this study, the highest number of QTLs was found in the B genome, with 30 QTLs (50%); 17 (28%) and 13 (22%) QTLs were found in genomes A, B and D, respectively (Table 3). Of these, ranging from 4 to 14 QTLs for each trait. They were mapped to 16 chromosomal locations with 1–3 QTLs per cluster, the largest gene cluster was located between 100008884|F|0 and 1237294|F|0 on the 6D chromosome (Fig. 2).

**Table 3:** Quantitative trait loci for mineral nutrient concentrations and agronomic traits in RILs population.

Trait	Environment	QTL name	Chromosome	Position	Confidence interval (cM)	LeftMarker	RightMarker	LOD	PVE(%)	Add
GZnC	E1	*QGZn.co-2B*	2B	517	516.5–517.5	2303802	2275590	2.77	7.12	1.78
	E1	*QGZn.co-6B.1*	6B	278	277.5–278.5	1252668	100005882|F|0	3.56	4.20	1.38
	E2	*QGZn.co-1B*	1B	383	382.5–383.5	1244708	3028438|F|0	2.76	5.35	−1.39
	E2	*QGZn.co-3A*	3A	100	99.5–100.5	3022261	3936326	3.56	5.18	2.45
	E2	*QGZn.co-4B*	4B	121	120.5–124	2277812	1242543	3.33	7.21	1.61
	E2	*QGZn.co-7A*	7A	286	285.5–288.5	5356706	5325178|F|0	5.47	7.83	1.81
	E3	*QGZn.co-3B*	3B	316	315.5–316.5	1002594|F|0	1103633	2.81	3.56	−0.85
	E3	*QGZn.co-3D*	3D	125	123.5–126.5	1372776	100008980|F|0	2.84	2.71	−2.65
	E3	*QGZn.co-5A*	5A	54	53.5–54.5	1244217	1272027|F|0	2.69	14.22	1.73
	E3	*QGZn.co-6B.2*	6B	180	177.5–180.5	3941131	990183	3.36	4.07	2.81
GFeC	E1	*QGFe.co-2A*	2A	147	146.5–147.5	4993302|F|0	3954215	4.12	3.00	2.77
	E2	*QGFe.co-1A*	1A	182	179.5–184.5	100008269|F|0	1134239|F|0	2.56	2.23	0.67
	E2	*QGFe.co-3B.1*	3B	200	199.5–200.5	1089107	1127875|F|0	3.65	14.56	−1.71
	E2	*QGFe.co-3D*	3D	125	124.5–125.5	1372776	100008980|F|0	2.57	2.23	−2.37
	E2	*QGFe.co-4B*	4B	123	120.5–124.0	1242543	1125612|F|0	5.33	4.41	0.93
	E2	*QGFe.co-5A.1*	5A	45	43.5–46.5	14394095|F|0	4543804	2.66	2.10	0.73
	E3	*QGFe.co-3B.2*	3B	263	262.5–263.5	1233878	4262223|F|0	3.14	5.62	2.67
	E3	*QGFe.co-5A.2*	5A	97	96.5–97.5	1102433	988523	3.09	6.94	2.09
	E3	*QGFe.co-6B*	6B	295	292.5–295.5	5332918	7342703	3.21	6.22	−0.73
GPC	E2	*QGpc.co-2A*	2A	306	302.5–308.5	1267600	1138191	3.53	4.22	0.18
	E2	*QGpc.co-2B.2*	2B	517	516.5–518.5	2303802	2275590	2.76	10.84	0.28
	E2	*QGpc.co-4A*	4A	3	0–18.5	3942314	5323574|F|0	2.78	3.16	0.15
	E3	*QGpc.co-2A*	2A	306	302.5–308.5	1267600	1138191	2.77	5.76	0.17
	E3	*QGpc.co-2B.1*	2B	310	304.5–312.5	1083804	1117983	3.09	6.33	−0.17
HD	E1	*QHD.co-6D*	6D	66	64.5–68.5	1209290	100008884|F|0	3.78	7.97	−2.36
	E2	*QHD.co-1A*	1A	93	92.5–93.5	994164	3023688	4.02	7.99	5.03
	E2	*QHD.co-3B*	3B	344	343.5–344.5	1152422	3936319	2.68	5.21	−3.27
	E2	*QHD.co-6D*	6D	67	65.5–68.5	100008884|F|0	1237294|F|0	3.12	6.40	−1.04
	E3	*QHD.co-1B*	1B	332	331.5–332.5	7940846|F|0	3950917	2.58	5.27	1.38
	E3	*QHD.co-6D*	6D	67	65.5–68.5	100008884|F|0	1237294|F|0	4.86	6.63	−1.68
MD	E1	*QMD.co-2B*	2B	41	40.5–41.5	1023861	7169514	3.24	3.89	−6.55
	E1	*QMD.co-3B.1*	3B	202	201.5–202.5	1090626	1718514	3.08	3.21	−2.91
	E1	*QMD.co-6B*	6B	170	169.5–170.5	5583068	1120240	3.85	7.56	3.43
	E1	*QMD.co-6D*	6D	67	65.5–68.5	100008884|F|0	1237294|F|0	4.96	4.24	−1.11
	E1	*QMD.co-7D*	7D	194	190.5–197.5	3950217	2372642	3.49	2.66	−0.78
	E2	*QMD.co-3B.2*	3B	344	343.5–344.5	1152422	3936319	8.07	12.28	−3.62
	E2	*QMD.co-4A*	4A	154	153.5–155.5	4398288	1228828|F|0	6.10	6.20	0.73
	E2	*QMD.co-6D*	6D	67	65.5–67.5	100008884|F|0	1237294|F|0	5.32	5.94	−0.80
	E2	*QMD.co-7B*	7B	78	77.5–78.5	987928	1052236	2.72	3.66	−1.28
	E3	*QMD.co-2B*	2B	41	40.5–41.5	1023861	7169514	4.09	4.86	−6.13
	E3	*QMD.co-3B.2*	3B	344	343.5–344.5	1152422	3936319	3.02	5.75	−3.37
	E3	*QMD.co-6D*	6D	67	65.5–67.5	100008884|F|0	1237294|F|0	7.94	7.40	−1.23
PH	E1	*QPH.co-2A*	2A	34	33.5–35.5	1010905	1109890	2.79	5.12	−1.01
	E2	*QPH.co-3B.1*	3B	130	128.5–130.5	4407876	1092573	4.46	5.74	−1.53
	E2	*QPH.co-3B.2*	3B	160	159.5–162.5	3028449	3028616|F|0	6.52	8.78	−1.89
	E1	*QPH.co-3B.3*	3B	197	196.5–197.5	5009687|F|0	3937454	2.72	9.94	1.41
TKW	E1	*QTKW.co-1A*	1A	132	131.5–132.5	1160755	1165294	2.53	13.56	−1.59
	E1	*QTKW.co-2A*	2A	291	288.5–292.5	3033455	2288824	5.97	6.27	−1.10
	E1	*QTKW.co-2B*	2B	524	523.5–524.5	3939679	1234002	5.03	5.12	1.02
	E1	*QTKW.co-3B*	3B	303	302.5–303.5	988768	1099934	3.00	3.36	3.55
	E1	*QTKW.co-5B.1*	5B	287	281.5–294.5	100005844|F|0	2322388	2.91	4.93	−1.06
	E1	*QTKW.co-5B.2*	5B	344	343.5–344.5	1165997	5323590|F|0	4.23	5.04	2.23
	E1	*QTKW.co-6D.1*	6D	61	51.5–63.5	4911065|F|0	1209290	2.73	3.24	−0.79
	E1	*QTKW.co-6D.2*	6D	68	66.5–69.5	100008884|F|0	1237294|F|0	4.47	5.99	1.16
	E2	*QTKW.co-2B*	2B	524	523.5–525.5	3939679	1234002	6.80	12.30	1.55
	E2	*QTKW.co-5A*	5A	220	214.5–225.5	100003298|F|0	1165921	3.65	6.36	−1.07
	E2	*QTKW.co-6D.2*	6D	67	65.5–69.5	100008884|F|0	1237294|F|0	3.73	7.06	1.29
	E3	*QTKW.co-2B*	2B	524	523.5–524.5	3939679	1234002	7.38	13.22	1.48
	E3	*QTKW.co-5A*	5A	220	214.5–225.5	100003298|F|0	1165921	3.75	6.42	−0.99
	E3	*QTKW.co-6D.2*	6D	67	65.5–69.5	100008884|F|0	1237294|F|0	3.73	7.01	1.18

Note: LOD likelihood of odds ratio for genetic effects, R^2^ total percentage of phenotypic variation explained (PVE) by each QTL, Add the additive effect.

#### 3.3.1 QTL analysis of GZnC

Ten QTLs, located on chromosomes 1B, 2B, 3A, 3B, 3D, 4B, 5A, 6B and 7A, had an positive effect on GZnC. These ten QTLs associated with a PVE (phenotypic variance explained) of between 2.71 and 14.22% (Table 3). For the QTLs located on chromosomes 1B, 3B and 3D, the Roelfs F2007 allele increased GZnC, whereas for the QTLs on 2B, 3A, 4B, 5A, 6B and 7A, the ‘Chinese parental line’ allele increased GZnC. The QTLs on chromosomes 3A, 3B and 3D were not in homoeologous position. The PVE for *QGZn.co-5A* was highest value (14.2%) across all environments, while the overall PVE for *QGZn.co-3D* was 2.71%.

#### 3.3.2 QTL analysis of GFeC

Nine QTLs on chromosomes 1A, 2A, 3B, 3D, 4B, 5A and 6B were detected for GFeC. The phenotypic variation explained by these QTLs ranged from 2.10 to 14.56%. For three of nine QTLs, the ‘Chinese parental line’ allele increased GFeC. The largest portion of the total phenotypic variation (R^2^ = 14.56%) was explained by *QGFe.co-3B.1* with an additive effect increase from the ‘Chinese parental line’ allele (Table 3).

#### 3.3.3 QTL analysis of GPC

Five QTLs were associated with GPC. These five QTLs associated with a PVE of between 3.16 and 10.84% (Table 3). For the QTLs located on chromosomes 2A, 2B, and 4A, the Roelfs F2007 allele increased GPC, whereas for the *QGpc.co-2B.1* on 2B, the ‘Chinese parental line’ allele increased GPC. *QGpc.co-2A* was the stably expressed QTL in E2, and also when performance was averaged across the all environments. The PVE for *QGpc.co-2A* was 5.76% across all environments. However, no QTL was detected in E1 environment.

#### 3.3.4 QTL analysis of TKW

Fourteen QTLs for TKW were identified in all, associated with a PVE of between 3.24 and 13.56% (Table 3). *QTKW.co-2B* was the most stably expressed QTL, followed by *QTKW.co-6D.2*. Both of these QTL were detectable in each of the two environments, and when performance was averaged across the two environments. The PVE for *QTKW.co-2B* was 13.22% across all environments, while the overall PVE for *QTKW.co-6D.2* was 7.01%. For the two QTLs, the Roelfs F2007 allele increased TKW. *QTKW.co-5A* was identified in E2 and across all environments. The *QTKW.co-1A* on chromosome 1A was detected in only one environment (E1), but the largest portion of the total phenotypic variation (R^2^ = 13.56%) was explained by *QTKW.co-1A*. In addition, other QTLs were located on chromosomes 2A, 3B, and 5B.

#### 3.3.5 QTL analysis of PH

Four QTLs on chromosomes 2A and 3B were detected for PH (Table 3). The phenotypic variation explained by these QTLs ranged from 5.12to 9.94%. *QPH.co-2A* mapping to chromosome 2A (flanked by the SNP marker 1010905 and 1109890) was detected in E1 environments and its PVE was 5.12% (Table 3). Other three QTLs mapping to chromosome 3B was detected in E1 and E2 environments, respectively. For *QPH.co-2A*, *QPH.co-3B.1* and *QPH.co-3B.2*, the ‘Chinese parental line’ allele played a role in reducing PH.

#### 3.3.6 QTL analysis of HD

A total of six QTLs were associated with HD. These six QTLs associated with a PVE of between 5.21 and 7.99% (Table 3). *QHD.co-6D* was the most stably expressed QTL in all environments and explained 7.97, 6.40 and 6.63% of the phenotypic variance, respectively. For this QTL, the ‘Chinese parental line’ allele delayed HD. Moreover, the other three QTLs were detected on 1A, 1B and 3B. Finally, a QTL on chromosome 1A was detected in only E2 environment, but the largest portion of the total phenotypic variation (R^2^ = 7.99%) was explained by *QHD.co-1A*. For *QHDco-1A* the Roelfs F2007 allele delayed heading date HD.

#### 3.3.7 QTL analysis of MD

Twelve QTLs on chromosomes 2B, 3B, 4A, 6B, 6D, 7B and 7D were detected for MD (Table 3). The phenotypic variation explained by these QTLs ranged from 2.66 to 12.28%. *QMD.co-6D* was the most stably expressed QTL in all environments and explained 4.24, 5.94 and 7.40% of the phenotypic variance, respectively. For this QTL, the ‘Chinese parental line’ allele delayed MD. The *QMD.co-3B.2* on 3B explained 12.28 and 5.75% of the phenotypic variation in E2 and E3, respectively. For *QMD.co-6B* and *QMD.co-4A*, the ROELFS F2007 allele showed a delayed effect on MD.

### 3.4 Pleiotropic and multigenic effects revealed by QTL mapping

Some of the genomic regions identified were found to be associated with more than one trait based on multi-trait QTL mapping. Of these, two genomic regions on 3B and 6D were simultaneously associated with HD and MD. This is consistent with the significant positive correlation between HD and MD (Fig. 2). In particular, the genomic region on 6D was not only significantly associated with HD and MD, but associated with TKW. The pleiotropic effects were supported by correlations between agronomic traits.

The GZnC QTL on chromosome3D was found at the same location as the QTL for GFeC. Moreover, genomic region on chromosome 2B was simultaneously associated with GZnC and GPC, which was closer to the genomic region associated with TKW (Fig. 2). Multigenic effects were also observed in this study, where each of traits GZnC, GFeC, GPC, PH, MD, HD and TKW was significantly associated with multi-trait markers (Table 3, Fig. 2).

### 3.5 Candidate genes that may be linked to traits

The sequence of the flanking marker of each QTL were entered in the International Wheat Genome Sequencing Consortium database (IWGSC; http://www.wheatgenome.org/) and Ensambl Plants database of the bread wheat genome sequence (http://plants.ensembl.org/index.html). The results showed that a total of 55 QTLs appeared to be located in regions were genes coded for diverse proteins, including mainly: .*cytochrome P450, Leucine-rich repeat receptor-like protein kinase, Protein kinase family protein, Acid phosphatase/vanadium-dependent haloperoxidase-related protein, protein embryonic flower 1,* etc (Table S2).

## 4 Discussion

The QTL mapping analysis is a useful tool to identify genomic regions associated with mineral nutrient concentrations and yield component traits. In addition, the validated QTLs and associated markers can be integrated into the elite germplasm through marker assisted breeding.

### 4.1 QTLs for mineral nutrient concentrations traits

GZnC, GFeC and GPC are quantitatively inherited traits, as shown by their continuous distribution across the RIL population (Fig. 1). Our results indicated that the RILs also showed some transgressive segregation in both directions suggesting that both parents carried a few different genes with alleles contributing to increased GZnC, GFeC and GPC, and this is in accordance with the previous results presented by others (Xu et al., 2012). A large number of previous QTL mapping studies have also mapped QTL for GZnC, GFeC and GPC in 21 chromosomes of wheat and wheat related species (Peleg et al., 2009; Krishnappa et al., 2017). In our study the largest PVE (14.56%) was displayed by *QGFe.co-3B.1* on chromosome 3B, followed by *QGZn.co-5A* with PVE 14.22% on chromosome 5A. In addition, some co-location of QTLs occurred for the three traits on chromosomes 2B (517 cM) and 3D (125 cM) (Fig. 2). Among them, QTL was detected for the GZnC and GPC on chromosomes 2B and another QTL was detected for GZnC and GFeC on chromosomes 3D. On chromosome 2B, the favourable parent allele was contributed by ‘Chinese parental line’ for GPC and GZnC. On chromosome 3D, the favourable allele was from Roelfs F2007 for the GZnC and GFeC. These genetic regions may have pleiotropic effects. Meanwhile, significant and positive correlations among GZnC, GFeC and GPC were found in this study, which was consistent with the studies in wheat (Xu et al., 2012; Zhao et al., 2009). All the results indicated common physiological and/or genetic basis for GZnC, GFeC and GPC in wheat, suggesting that these three traits could be improved simultaneously (Zhao et al., 2009).

The famous *Gpc-B1* gene on chromosome 6BS conferring stable expression of GPC, GZnC and GFeC from wild emmer has been cloned (Uauy et al., 2006a). In this study, the highly associated markers were used to predict putative candidate genes for QTLs associated with mineral nutrienttraits. For the two traits, the results showed that on chromsomes 1A, 1B, 2A, 2B, 3A, 3B, 3D, 4A, 4B, 5A, 6B and 7A there are genes encoding for the *cytochrome P450*, *acid phosphatase*, *tyrosine decarboxylase*, *zinc-binding in reverse transcriptase*, *GRF zinc finger protein*, *auxin response factor*, *heavy metal-associated domain containing protein*, *protein kinase* and so on (Table S2) Among them, the *cytochrome P450* is reported to be related to Zn and Fe homeostasis, and frequently expressed under high Zn conditions in Arabidopsis (van de Mortel et al., 2006). The *heavy metal-associated domain containing protein*, *zinc-binding in reverse transcriptase*, *GRF zinc finger protein* and *acid phosphatase* may play major roles in accumulating additional Zn and Fe to wheat grain. The *QGZn.co-5A* on chromosome 5A displayed a large PVE 14.22% across two years, the BLAST results showed that there are genes encoding for the *FAR1 protein* and *zinc-binding in reverse transcriptase*, which are related with zinc ion binding (Hudson et al., 2003). The *QGpc.co-4A* displayed its location in a region where genes code for the *protein kinase* and, which are reported to be involved in catalyze phosphorylation processes in which some protein structures are Zn related (Scheeff and Bourne, 2005). Furthermore, for the *QGFe.co-3D* and *QGZn.co-3D* on the same location, the BLAST results showed that on chromosome 3D there are genes encoding for the *retrotransposon protein*, which is related with zinc-binding in reverse transcriptase. The *QGZn.co-2BQ* and *Gpc.co-2B.2* displayed its location in a same region on chromosome 2B where genes code for the *purple acid phosphatase*, *NBS-LRR disease resistance protein-like protein* and *auxin response factor*.

### 4.2 QTLs for agronomic-related traits

Four QTLs for PH were mapped on chromosomes 2A and 3B (Fig. 2). These QTLs mapped in the similar locations of chromosomes 2A and 3B were also reported by Cui et al. (2011). Our BLAST results showed that candidate genes related to PH are involved in *cysteine-rich receptor kinase* (*CRK*), *hydrolases superfamily protein* and *NBS-LRR resistance-like proteinetc* (Table S2). Some related studies have demonstrated that *CRK5* affects plant growth and development in Arabidopsis, which further proves that this gene is closely related to PH (Burdiak et al., 2015).

Of four QTLs detected for HD, one QTL on chromosomes 6D was coincident with QTLs for MD and TKW, and another QTL on chromosome 3B was located in the same position as the QTL for MD (Fig. 2). This is consistent with our results that HD was significantly correlated with MD. Candidate genes associated with the multiple QTLs on chromosomes 3B is involved in *protein embryonic flower 1* (*EMF1*) and *auxin response factor 11* (Table S2). The former researches have indicated that the plant-specific *protein embryonic flower1* (*EMF1*) functions in maintaining the repression of the flower homeotic gene AGAMOUS (AG) during vegetative development in *Arabidopsis thaliana* by acting in concert with the *EMF2* complex (Calonje et al., 2008). Wang et al. (2014) showed that plant-specific embryonic flower1 (*EMF1*) is involved in promoting Arabidopsis flowering under long daylight. Another multiple QTLs on chromosomes 6D displayed its location in a region where genes code for the *protein kinase family protein*, *endonuclease/exonuclease/phosphatase* and *UDP-glucosyl transferase* (Table S2). Previous related research showed that the expression abundance of *UDP-glucosyl transferase* in the starch synthesis pathway is different during the endosperm filling period (Yamakawa et al., 2007). Furthermore, putative *protein phosphatase* with kelch-like repeat domain is related to grain length, and usually thousand kernel weight is positively correlated with grain length, suggesting that *phosphatase* is also related to TKW (Zhang et al., 2012). The stable *QTKW.co-2B* was located to a QTL for thousand kernel weight on chromosome 2B also identified by Ramya et al. (2010) in a bread wheat population.

## 5 Conclusions

In conclusion, few QTLs for mineral nutrient concentrations and yield componenttraits were co-located, indicating that there is a great opportunity for simultaneous selection which was evident from the significant positive correlation between GPC and GZnC, GZnC and GFeC. Although there was a significant positive correlation between GPC and TKW, but no co-location QTL was found in this study. Meanwhile, the mineral nutrient concentrations of the RIL population were significantly improved compared to the parents, and these results suggested that the rich genetic diversity for mineral nutrient concentrations in landraces provides novel alleles for genetically enhancing wheat grain mineral nutrient concentrations. The promising QTLs identified for GZnC, GZnC and GPC offers further possibilities for selective biofortification breeding of wheat with enhanced GZnC and GFeC. The major and intermediate effect QTLs from diverse sources were effectively utilized in Biofortification breeding program by pyramiding QTL regions through marker assisted breeding.

## Conflicts of interest

Authors declare there are no conflicts of interest.
