# Supplementary material for: QTL mapping for micronutrients concentration and yield component traits in a hexaploid wheat mapping population
Source: J Cereal Sci. 2019 Jul;88:57–64. doi: 10.1016/j.jcs.2019.05.008 (PMC7729826; doi:10.1016/j.jcs.2019.05.008)
Supplement: Multimedia component 2 [file mmc2.docx]

Table S1.Construction of genetic map markers distribution in the A,B,D,and whole genomes.

| Chr. | No.markers | | | | Map length(cM) | | | | Marker density(cM/marker) | | | |
| --- | --- | --- | --- | --- | --- | --- | --- | --- | --- | --- | --- | --- |
|  | A | B | D | Total | A | B | D | Total | A | B | D | Total |
| 1 | 119 | 520 | 68 | 707 | 187 | 470 | 135 | 792 | 1.6 | 0.9 | 2.0 | 1.1 |
| 2 | 205 | 323 | 81 | 609 | 403 | 577 | 229 | 1209 | 2.0 | 1.8 | 2.8 | 2.0 |
| 3 | 166 | 336 | 41 | 543 | 228 | 402 | 212 | 842 | 1.4 | 1.2 | 5.2 | 1.6 |
| 4 | 137 | 44 | 77 | 258 | 255 | 124 | 116 | 495 | 1.9 | 2.8 | 1.5 | 1.9 |
| 5 | 127 | 326 | 28 | 481 | 246 | 413 | 168 | 826 | 1.9 | 1.3 | 6.0 | 1.7 |
| 6 | 190 | 204 | 43 | 437 | 232 | 349 | 134 | 715 | 1.2 | 1.7 | 3.1 | 1.6 |
| 7 | 254 | 202 | 65 | 521 | 326 | 334 | 208 | 867 | 1.3 | 1.7 | 3.2 | 1.7 |
| All | 1198 | 1955 | 403 | 3556 | 1876 | 2670 | 1200 | 5747 | 1.6 | 1.4 | 3.0 | 1.6 |

Note: Chr, Chromosome; cM, centimorgan.

Table S2. List of candidate genes associated with mineral nutrient concentrations and agronomic traits.

| QTL | Chr. | Left marker | Sequence left marker | Right marker | Sequence right marker | Candidate genes(closest/nearby) |
| --- | --- | --- | --- | --- | --- | --- |
| *QGFe.co-1A* | 1A | 100008269\|F\|0 | TGCAGAAGGCTCTTGTATTATTGCATCGGGCCATCGGCAGCTCACCATCAGCCGAGATCGGAAGAGCGG | 1134239\|F\|0 | TGCAGGAGGCAGCCGACGACGCACTGGTAAAAAAAGAACCTATAGTCCCGAGATCGGAAGAGCGGTTCA | Acid phosphatase |
| *QGFe.co-2A* | 2A | 4993302\|F\|0 | TGCAGCAGCCAAATTCAAAATCCGGACTAACATACCTGGAGTGTCCGAGATCGGAAGAGCGGTTCAGCA | 3954215 | TGCAGCTGCATCTCCAACACGGGTCACTAAAACGTCCTCAAATGTCCGAGATCGGAAGAGCGGTTCAGC | Cytochrome P450 family protein |
| *QGFe.co-3B.1* | 3B | 1089107 | TGCAGGAAGGACTCAGTGGTGGTGTTGAATACATGGATAATGCTGCCCGTGCACCAATGGAGGCTACCC | 1127875\|F\|0 | TGCAGGCACACTTGCTTGGCCAGGGGAGAAGCCCGTACGTGGACCGCGACGCACAGACGACCGCGGCAA | Leucine-rich repeat receptor-like protein kinase |
| *QGFe.co-3B.2* | 3B | 1233878 | TGCAGGCCTGACTTCCAGCAGTCCGAAGGCTCTGTCCTCACAAGGTGGTGATGCAAAGCTGGTGACCGA | 4262223\|F\|0 | TGCAGCTTGCTGGTTGGATCACAGCGTCGACGCAGCCGAGATCGGAAGAGCGGTTCAGCAGGAATGCCG | Beta-fructofuranosidase |
| *QGFe.co-4B* | 4B | 1242543 | TGCAGCAGCTTTTAAGCGTGCACTGCTCCGTATGCACACACGCGATATCAGCTTTCAACCTGATGTTCA | 1125612\|F\|0 | TGCAGCCGTACGTCACGCCAACATGAGGAGATGCGTACGTATCAGGCAACAAAAAGACCACCAAAAGCT | Cytochrome P450 |
| *QGFe.co-5A.1* | 5A | 14394095\|F\|0 | TGCAGGCAGCCCCACGGCCTCCCGAGATCGGAAGAGCGGTTCAGCAGGAATGCCGAGACCGATCTCGTA | 4543804 | TGCAGGCCGCCATGCAGCCTGACGTGGACGCCGAGATCGGAAGAGCGGTTCAGCAGGAATGCCGAGACC | zinc finger protein 4 |
|  |  |  |  |  |  | Tyrosine decarboxylase |
| *QGFe.co-5A.2* | 5A | 1102433 | TGCAGCGCAGCAGGAGAAAGATGAGTGTATTTCTCGTCGCAGCCCTGGTGGCCTGCCTTGTCAGCCATG | 988523 | TGCAGCATCAACCTCATTTGCAGATCCATCGGCTAGCTACTTCTCAAGATCCGAGATCGGAAGAGCGGT | P-loop containing nucleoside triphosphate hydrolases superfamily protein |
| *QGFe.co-6B* | 6B | 5332918 | TGCAGCCGAGGCTTCAATGGACAGCGTACGGCTCCGAGATCGGAAGAGCGGTTCAGCAGGAATGCCGAG | 7342703 | TGCAGTCTACACCTCGCGCCATGGCCTCCACTTCGACCTCTCCTGCTGCTGCTGGCCTGGGTATAGTTT | F-box protein |
| *QGpc.co-2A* | 2A | 1267600 | TGCAGTGTTGACAGAGCGATGGACTTAGCAGCTAAGAAGAGAAAGGCTTCCCAGAGGCAACAACATGAG | 1138191 | TGCAGCAGCAGCAGCAGCACCAGGCTCTATCAGGCCATCATCTTCAGAAACTGTATCGGAATCTGCTTG | Protein FAR1-related sequence 6 |
| *QGpc.co-2B.1* | 2B | 1083804 | TGCAGGCGCTGGCTAGATACTGGCGCTCTTACGCCTGGCGTAGTGGGCCGAGATCGGAAGAGCGGTTCA | 1117983 | TGCAGCAACATGGGGAGGGAGCAACTGCTGCGTGCATATGCAGGAGCAGCAACAAGCAACCGAGATCGG | Heavy metal-associated domain containing protein |
| *QGpc.co-4A* | 4A | 3942314 | TGCAGCTCGGCGAAGTGCAGGATAAAGATGCACCCCGAGATCGGAAGAGCGGTTCAGCAGGAATGCCGA | 5323574\|F\|0 | TGCAGCGATGCAGGTGGTGCCGCTGCCGAGATCGGAAGAGCGGTTCAGCAGGAATGCCGAGACCGATCT | Protein kinase |
|  |  |  |  |  |  | Photosystem II reaction center W protein |
| *QGZn.co-1B* | 1B | 1244708 | TGCAGCAACGCTCCTTCTTCGCCCATACCGACAGCTCCAGGTCCTCGGCCCGAGATCGGAAGAGCGGTT | 3028438\|F\|0 | TGCAGGTACATTTGCCGGAGGAGCTGCACCATCACACAGTCGCCGAGATCGGAAGAGCGGTTCAGCAGG | Endoribonuclease YbeY |
| *QGZn.co-2B*/*QGpc.co-2B.2* | 2B | 2303802 | TGCAGTGATTGGAGCCATGATGACTAATGACTCAACGGCGATGTCGATGGCCGCAGCGACGGCGACGAT | 2275590 | TGCAGGGCTACACAAGCTTATCAACCTCAAGAAACTAACTGTCGATCAGTGTTCGTCCATCCGAGATCG | Purple acid phosphatase |
|  |  |  |  |  |  | NBS-LRR disease resistance protein-like protein |
|  |  |  |  |  |  | Auxin response factor |
| *QGZn.co-3A* | 3A | 3022261 | TGCAGCAGTACGTCCTGTTTGATGCCCTCCATGACCCGAGATCGGAAGAGCGGTTCAGCAGGAATGCCG | 3936326 | TGCAGGGAGGGCGGCTATCATTGTCAGTCGGATCCCGAGATCGGAAGAGCGGTTCAGCAGGAATGCCGA | Cytochrome P450 |
| *QGZn.co-3B* | 3B | 1002594\|F\|0 | TGCAGCGGCGCATCAATGGCGTGAGAACGACTGGAAGAGACATAGCAGCATCAGCATCAGGGTAGCAGC | 1103633 | TGCAGATCGGCACCCCTTTGTGACTCCTGATCGTACGCGGCAGTTACATGGAACAGCCCCCAAGGAGCC | Serine/threonine protein phosphatase 7 long form isogeny |
| *QGZn.co-3D*/*QGFe.co-3D* | 3D | 1372776 | TGCAGCACGAAGGCGATCAGTGTAGGCGCCCTCATCATGACCTCGTTCATCTCGATCCTCCGAGATCGG | 100008980\|F\|0 | TGCAGCTTCCAGACACAAGTGGACGCGCGAGTGCGCGTGGAAAAGCCGTGGATGCCGAGATCGGAAGAG | Retrotransposon protein ( zinc-binding in reverse transcriptase) |
| *QGZn.co-4B* | 4B | 2277812 | TGCAGTGCCCCGACCTCCACGCATCAGCACCTCTGCATCGACCAGGCCACCGAGAGCCCCTGCGCACGC | 1242543 | TGCAGCAGCTTTTAAGCGTGCACTGCTCCGTATGCACACACGCGATATCAGCTTTCAACCTGATGTTCA | Double Clp-N motif-containing P-loop nucleoside triphosphate hydrolases superfamily protein |
| *QGZn.co-5A* | 5A | 1244217 | TGCAGAGCAGCAGCCCAATCATGTGGGCCTGCTAGCTGGCATGAAGTTAGGCAGCCGAGATCGGAAGAG | 1272027\|F\|0 | TGCAGGCGCTTGCATAGGCGGCAGTTGAGCAGTAGCGGCGCGGCGGCGCTGCTAGTGGCCGAGATCGGA | Protein FAR1 ( zinc ion binding) |
|  |  |  |  |  |  | zinc-binding in reverse transcriptase |
| *QGZn.co-6B.1* | 6B | 1252668 | TGCAGGCCGAGGCGGACGCCAAGCTGTTGAGGGAGCGGTGGATCCTGCTGGCGCACACGCACAAGACCG | 100005882\|F\|0 | TGCAGAAGCCTAGACGTACAAGACGACAGCGTGCACATATATATGGCTACAGGTGACAAACAGTACGTA | LRR family protein |
| *QGZn.co-6B.2* | 6B | 3941131 | TGCAGCGCGATGGCCTACGTCTTTCTCATCGTCTCCGAGATCGGAAGAGCGGTTCAGCAGGAATGCCGA | 990183 | TGCAGAATGATCATGGACTCGCAGACCGGGGCGCCGTTGTGGATGAGCACGGGTATCTTCTTGTGCACC | Glutathione S-transferase |
| *QGZn.co-7A* | 7A | 5356706 | TGCAGCCATCCAACGCGGCGGCGCGCCGTCGGCGGCGGCGGCGATGCACACGCAACCTATGGCTTCCGA | 5325178\|F\|0 | TGCAGACAAGGCAACCTCCATGGCCGAGATCGGAAGAGCGGTTCAGCAGGAATGCCGAGACCGATCTCG | GRF zinc finger protein |
|  |  |  |  |  |  | glycosyltransferase family protein |
| *QHD.co-1A* | 1A | 994164 | TGCAGGGCACCGTATAATTGATGGGCGCCGTATACGTGCGGGGGCTGCGGACGAAATCCATCTGGGGGC | 3023688 | TGCAGGCTGCCTTGTCCAAACTCCAGGCCAAGCACCGAGATCGGAAGAGCGGTTCAGCAGGAATGCCGA | Retrovirus-related Pol polyprotein from transposon TNT 1-94 |
| *QHD.co-1B* | 1B | 7940846\|F\|0 | TGCAGATTCCGGCCCAAAAGTTTGTCGGTCCGAGATCGGAAGAGCGGTTCAGCAGGAATGCCGAGACCG | 3950917 | TGCAGCCAAGGCCATAGCCAAGTGGGGCCGTCCGAGATCGGAAGAGCGGTTCAGCAGGAATGCCGAGAC | Chalcone synthase |
| *QHD.co-6D* | 6D | 1209290 | TGCAGAAGAAGAAGTTGAAGGCAGAGCTGTAAGCTTCTCCTTGGTTAGCTTCTCCGAGATCGGAAGAGC | 100008884\|F\|0 | TGCAGTTGCTGGTTAACCCTGCCAAAGTCCCGAGATCGGAAGAGCGGTTCAGCAGGAATGCCGAGACCG | Thioredoxin |
|  |  |  |  |  |  | Protein kinase family protein |
| *QMD.co-2B* | 2B | 1023861 | TGCAGGAAGAATTGACAAGTGCAGGTGCAACAGCCATTTACCTGAATATGTGAATCAGACCGAGATCGG | 7169514 | TGCAGCGGTTCCCGCGGGGCGGCGCCGAGATCGGAAGAGCGGTTCAGCAGGAATGCCGAGACCGATCTC | zinc finger MYM-type-like protein |
| *QMD.co-3B.1* | 3B | 1090626 | TGCAGCCAGATGGTCCCGACGAGGCTCAGCCAATGGGCCGAGGAAGGCGACGACATCGCCGAGGAGAGG | 1718514 | TGCAGGGACCTGGGCGATGAAGACTCCGACGAGCAGCGCCAGTGCAAGCGCCGAGATCGGAAGAGCGGT | Glycoside hydrolase family |
| *QMD.co-3B.2*/*QHD.co-3B* | 3B | 1152422 | TGCAGAACTCCACCAACGGGTGGATCATGGACTCAGGCGCTTCTTCTCACATTACCTCGGACCCAGGTA | 3936319 | TGCAGCAGTTCCGCTCCTACTTCCAAAGGGTAAACAGGAGTGCACCGAGATCGGAAGAGCGGTTCAGCA | Auxin response factor 11 |
|  |  |  |  |  |  | Protein embryonic flower 1 (EMF1) |
| *QMD.co-4A* | 4A | 4398288 | TGCAGGGCTCAGATGGCCGAAGCAGAGAGGCCGAGATCGGAAGAGCGGTTCAGCAGGAATGCCGAGACC | 1228828\|F\|0 | TGCAGCGAGAGATGAGACAGGCATATGAGATGGGAAGAGGGAGGGGAGGGACAGGCGGCGGAGGAGCTG | elongation factor family protein |
| *QMD.co-6B* | 6B | 5583068 | TGCAGATGAGGTGGACGGAGCGGAAGGGGCCCGAGATCGGAAGAGCGGTTCAGCAGGAATGCCGAGACC | 1120240 | TGCAGAGGTGGCGGTTTATAGCACAACTGTAGCAAAAAAGGGTACCAGGCGGCTCAAGAAGGGATCCTC | FBD-associated F-box protein |
| *QMD.co-7B* | 7B | 987928 | TGCAGGCAACATCATCTGCGTGGCTCGTCAACCGCTGGAGGATGAAGAGTTGAATGCTCGGCTCTAACA | 1052236 | TGCAGAGGCCTACTTCAATGAGCTAGTGAATAGGAGTATGATTGACCAAGCCGAGATCGGAAGAGCGGT | NBS-LRR disease resistance protein-like |
| *QMD.co-7D* | 7D | 3950217 | TGCAGGCACTGTCGCTCGTGCGCTTTCCAAGTGTACCCGAGATCGGAAGAGCGGTTCAGCAGGAATGCC | 2372642 | TGCAGGTGCTCTGCTTCTTGGTCGAGATGTGAAGGCTCGCGCTGGTTAGTGCAGTGAGATGGGTATGGT | seed storage 2S albumin-like protein |
|  |  |  |  |  |  | Pollen-specific protein |
| *QPH.co-2A* | 2A | 1010905 | TGCAGGATGTTGTCCGTGCTCCAGAGGCTGCCCTCGTCGGCGTCGTCGCAGCCACCGCAGTGGCTGAAA | 1109890 | TGCAGGATCATCAACTATCCTTACTTTTGAGGCTTCACCACAAGGCACCGAGATCGGAAGAGCGGTTCA | P-loop containing nucleoside triphosphate hydrolases superfamily protein |
|  |  |  |  |  |  | NBS-LRR resistance-like protein |
| *QPH.co-3B.1* | 3B | 4407876 | TGCAGTGGCGGAGCCAGCCCGTGAATGCAGCCCGAGATCGGAAGAGCGGTTCAGCAGGAATGCCGAGAC | 1092573 | TGCAGCGAGGTGCTTTTGGAGGAGGATAGGCAGGATTCAGACATCCAGCAGGAGATGGGGCTCAAGAAG | Protein kinase superfamily protein |
| *QPH.co-3B.2* | 3B | 3028449 | TGCAGGCTGGGAGAAGACAAGCCGCGCACATTTGTGCCGAGATCGGAAGAGCGGTTCAGCAGGAATGCC | 3028616\|F\|0 | TGCAGTAGATCAACGAGGAGGGCGCCACCACCACTGTTATCAAGCCGAGATCGGAAGAGCGGTTCAGCA | Cysteine-rich receptor kinase (CRK) |
| *QPH.co-3B.3* | 3B | 5009687\|F\|0 | TGCAGCAGCTCGATGTAGGTCGCTCGATGCGTTGGCGGCTGATGCCGAGATCGGAAGAGCGGTTCAGCA | 3937454 | TGCAGCGGCGCCAAGTTCCAACAAGGACTGAATGTGTTTTCTGTGGCCGAGATCGGAAGAGCGGTTCAG | Retrotransposon protein |
| *QTKW.co-1A* | 1A | 1160755 | TGCAGCAAACTGCCACTGACCTCGGCCGCTGCCATCTCGTACGACGCCGAGATCGGAAGAGCGGTTCAG | 1165294 | TGCAGGCTGCTCTTCACAGCGCCACCGCGGGGCCTTCGTCATCTGGCAGCACCTCTGACTGGTACTTGG | Retrovirus-related Pol polyprotein from transposon TNT 1-94 |
| *QTKW.co-2A* | 2A | 3033455 | TGCAGCAAGCTGGCGATGGAACGATGATGGAAAACGCCGAGATCGGAAGAGCGGTTCAGCAGGAATGCC | 2288824 | TGCAGGGACATGGACAACATGGAGGTCGTCGTGCTCGACCCCGACAGCAACAACCGAGATCGGAAGAGC | Glutathione S-transferase T3 |
| *QTKW.co-2B* | 2B | 3939679 | TGCAGTAAGGACCTGGCCGAATCGCTTCCTCGTAGCTGCCCGAGATCGGAAGAGCGGTTCAGCAGGAAT | 1234002 | TGCAGACAGTGCATGGTGTGAAGCCCCACCAAGGGGAATGGGACCAGCTCAACTGCCCGCTGCTCAAGA | Disease resistance protein (NBS-LRR class) |
| *QTKW.co-3B* | 3B | 988768 | TGCAGGTGGCAATGGGGATGGTAAAGCAGACGAAGAGCGAGGGGACTTGTCCGAGATCGGAAGAGCGGT | 1099934 | TGCAGTGTACATGGAATAGCGCGGTTTATGGTCACATCAGATATCCATTCCCCGAGATCGGAAGAGCGG | bZIP transcription factor |
| *QTKW.co-5A* | 5A | 100003298\|F\|0 | TGCAGAGGGAAAGTTATTACCACCCGAGATCGGAAGAGCGGTTCAGCAGGAATGCCGAGACCGATCTCG | 1165921 | TGCAGGCCCCGCGTTGGCGGCGGCGGAGGCGGAGGAGGAGCGGCGCTGTCGCGCTGATCCGCCGAGATC | FAR1-related sequence protein |
|  |  |  |  |  |  | Auxin responsive protein/Disease resistance protein (NBS-LRR class) family |
| *QTKW.co-5B.1* | 5B | 100005844\|F\|0 | TGCAGAGAGCGCGCTGTCGGATTTGACGCCGGCGAGCAGAAAGCACAGGAGGTCGATCCAGTTCCGAGA | 2322388 | TGCAGTCCCGAATCGAACCCTGCTGACAGACACTAGCAGCAGAACTAGATCACTGTGCCGAGATCGGAA | Peptidase C19 |
| *QTKW.co-5B.2* | 5B | 1165997 | TGCAGCTCACAGACAGGCTGCTTGCTGTCCATCTATCATCGGGTACTCCGAGATCGGAAGAGCGGTTCA | 5323590\|F\|0 | TGCAGCGGGCCGCCACGATTGGGGAGGCGGGTGGCGGCGCCGAGATCGGAAGAGCGGTTCAGCAGGAAT | Retrotransposon protein (zinc-binding in reverse transcriptase) |
| *QTKW.co-6D.1* | 6D | 4911065\|F\|0 | TGCAGGTTGCTTCGTCCATCACCGAGATCGGAAGAGCGGTTCAGCAGGAATGCCGAGACCGATCTCGTA | 1209290 | TGCAGAAGAAGAAGTTGAAGGCAGAGCTGTAAGCTTCTCCTTGGTTAGCTTCTCCGAGATCGGAAGAGC | \ |
| *QTKW.co-6D.2*/*QHD.co-6D*/*QMD.co-6D* | 6D | 100008884\|F\|0 | TGCAGTTGCTGGTTAACCCTGCCAAAGTCCCGAGATCGGAAGAGCGGTTCAGCAGGAATGCCGAGACCG | 1237294\|F\|0 | TGCAGGCGGTATGCAGCTCTGCTATCCCGTGGTTCCAACTCTGACGGGTCACGGTCCTGGCTTTGCTCA | Endonuclease/exonuclease/phosphatase |
|  |  |  |  |  |  | UDP-glucosyl transferase |

Note: Chr, Chromosome.
